# Ultra-processed food consumption and its relationship with nutrient intake and dietary patterns in adolescents

**DOI:** 10.3389/fnut.2026.1748813

**Published:** 2026-04-09

**Authors:** Anwar H. AlBaloul

**Affiliations:** Department of Community Medicine and Behavioral Sciences, Faculty of Medicine, Kuwait University, Kuwait City, Kuwait

**Keywords:** adolescents, dietary quality, NOVA classification, nutrient intake, obesity in Kuwait, ultra-processed foods

## Abstract

**Background:**

Global population growth has increased food demand, leading to greater reliance on processed foods, which now account for about 75% of global food sales. Food processing transforms raw ingredients to improve safety, preservation, and sensory qualities. While processing supports food security, high consumption of ultra-processed foods (UPFs) is linked to poorer diet quality, higher intake of sugars and fats, lower intake of fiber and protein, and increased risks of obesity and obesity-related diseases.

**Objective:**

The study aims to assess the association between UPF consumption and dietary quality among adolescents in Kuwait to inform strategies for improving nutrition and reducing chronic disease risk.

**Methods:**

A cross-sectional study of 375 Kuwaiti adolescents assessed dietary intake using MyFood24 Middle East and evaluated diet quality via the Children’s Eatwell Guide. Foods were classified by the NOVA system, and associations between ultra-processed food intake and diet quality were analyzed using adjusted linear regression models.

**Results:**

Adolescents in Kuwait consumed a median of 443 g/day of UPFs, contributing 43.9% of total daily energy intake. Higher UPF consumption was associated with lower protein and fiber intake, and higher intakes of total fat, saturated fat, trans fats, and salt, indicating poorer overall diet quality.

**Conclusion:**

Greater UPF intake among Kuwaiti adolescents is linked to an unbalanced nutrient profile that may increase the risk of obesity and metabolic disorders. These findings highlight the need for targeted public health strategies to promote healthier dietary patterns in this population.

## Background

The global population reached approximately 8.2 billion in 2024 and is projected to continue growing over the coming decades, placing increasing demand on food systems (1). This growth has been directly associated with an increase in food demand. The United States Department of Agriculture (USDA) reported that the global agricultural food production has increased by 61%, based on the total calories available for human consumption (2). Food technology and processing enhance the efficiency, safety, and shelf life of available food. This may help with meeting the global demand by reducing waste and extending shelf life. As a result, approximately 75% of food sales worldwide now consist of processed foods to meet the demand of the modern food systems in high-income countries, although this estimate does not include minimally processed or unprocessed foods (3). In the Gulf and broader MENA region, the availability and consumption of UPFs are also increasing, reflecting the growing influence of modern food systems on adolescents’ dietary patterns (4).

Food processing is the application of operational transforms (physical, chemical, or biological techniques) to raw agricultural products into food for consumption (5). Processing aims to change the form of food, preserve it, enhance its safety, and improve its sensory properties. Food processing is classified based on how much the raw food is transformed, ranging from simple preparation to highly industrialized and often heavily modified products (6). Several systems have been proposed to classify foods based on their degree of processing (7, 8). The NOVA classification is the most widely used system, and it is currently the only one that specifically defines and categorizes ultra-processed foods (UPFs) (9). The NOVA system classifies all foods into four groups: unprocessed food (NOVA 1), processed culinary ingredients (NOVA 2), processed food (NOVA 3) and ultra-processed food (NOVA 4) (10). Monteiro et al. defined UPFs as industrial formulations made entirely or mostly from substances extracted from food (such as fats, oils, sugars, starches, and proteins), or synthesized in laboratories from food substrates or other organic sources, including food additives and flavorings (10). Several highly consumed food products are classified as UPF, including sweet and savory snacks (crisps, candy, and biscuits); sugary drinks and energy drinks; mass-produced breads, pastries, and cakes; ready meals such as frozen pizzas and instant noodles; and processed meats like sausages and chicken nuggets (10).

Food processing is rapidly growing, which is a positive sign regarding meeting the global food demand. However, whether that translates into better outcomes depends on how processed food fits into the overall dietary pattern. Adolescents are particularly vulnerable to dietary exposures, and high UPF consumption during this critical period may affect both diet quality and long-term health outcomes. A meta-analysis of observational studies including children and adolescents from multiple countries and diverse economic settings showed that higher UPF consumption was associated with increased intake of free sugars, total fats, and saturated fats, along with decreased intake of dietary fiber and protein (11). Additionally, the same study proposed that UPFs are consumed at the expense of unprocessed foods (11). Such an unhealthy dietary pattern was found to be positively associated with cardiovascular risk (12), breast cancer (13), and overweight and obesity (14). This evidence highlights the need to effectively assess the associations between UPFs and the overall dietary pattern and health outcomes, among various populations and age groups.

Adolescence is a critical period of growth and development during which nutritional adequacy is essential for supporting both physical maturation and cognitive function (15). Recently, adolescence has been identified as a critical window to assess nutritional intake and to prevent future nutrition-related diseases (16, 17). Adolescents’ dietary habits are shaped by early taste formation and reinforced over time, with exposure to UPFs influencing life-course dietary patterns (18). Structural determinants such as marketing, availability, and affordability further increase their vulnerability to high UPF intake, highlighting why this age group is particularly susceptible to poor dietary quality and unhealthy eating behaviors (19, 20). In the USA, it is estimated that UPF contribute 67.7% of the total caloric intake of adolescents ages 12–19 (21). In the UK, the average energy intake from UPFs is estimated as 65.9% (22).

Recent studies report that 52.1% of adolescents in Kuwait are either overweight or obese (23), while local data indicate that 46.4% of obese adolescents are prediabetic (24). This alarming trend underscores the potential role of adolescent obesity in driving the country’s increasing burden of non-communicable diseases (NCDs). High consumption of UPFs has been consistently associated with poorer dietary quality and a higher risk of obesity among adolescents. However, evidence from Middle Eastern populations remains limited. The present study seeks to address this gap by investigating the relationship between UPF consumption and dietary quality among Kuwaiti adolescents. Specifically, it aims to evaluate how UPF intake influences nutrient intake patterns and diet quality indices. A clearer understanding of these associations is essential for informing public health strategies and nutritional guidelines aimed at improving adolescent health and reducing the risk of diet-related chronic conditions.

## Materials and methods

This school-based cross-sectional study recruited a total of 375 adolescent participants, comprising both males and females aged 11–17 years, enrolled in high schools across all six governorates of the State of Kuwait. The minimum required sample size (*n* = 350) was calculated to ensure that the estimated sample proportion would fall within ±0.05 of the true population proportion, with a 95% confidence level.

Participants were selected using a multi-stage random sampling technique. In the first stage, schools were stratified by gender (boys’ and girls’ schools), reflecting the structure of Kuwait’s public education system. A random sampling procedure was then used to select schools across both gender strata. Kuwait has a total of 210 high schools distributed across six governorates, with the number of schools ranging from 14 in Mubarak Al-Kabeer to 29 in Al-Ahmadi, the governorate with the highest number. Furthermore, female enrollment in Kuwait’s public high schools exceeds that of males. To ensure representative sampling, a proportionally larger number of students were selected from Al-Ahmadi and a smaller number from Mubarak Al-Kabeer. Additionally, the sample included more female than male participants to reflect the gender distribution in the population.

In the second stage, academic grades and individual students were selected. Kuwaiti high schools comprise three academic grades. A random sampling approach was applied to select both the grades and the students within those grades. After the final sample was identified, students were invited to participate in the study. Written informed consent was obtained before inclusion.

This study’s inclusion criteria were based on adolescents aged 11–17 years without a prior diagnosis of chronic diseases, cardiovascular disease, diabetes, hypertension, or history of bariatric surgery, as these conditions could affect dietary intake. Only participants who provided written informed consent along with parental consent were enrolled.

### Anthropometric assessment

Anthropometric assessments included measurements of body weight, height, and waist circumference, conducted by trained researchers following standardized procedures. Body weight was measured to the nearest 100 g using calibrated portable digital scales, with participants wearing light clothing and no shoes. Height was measured to the nearest centimeter using a measuring tape, with participants standing upright without shoes. Waist circumference was measured using a non-elastic measuring tape, in accordance with World Health Organization (WHO) guidelines, at the midpoint between the lowest rib and the iliac crest (25). Anthropometric measurements were conducted in a private setting, with duplicate measurements for accuracy.

### Dietary data collection

Dietary intake data were collected using a single 24-h dietary recall administered during school visits using the MyFood24 dietary assessment tool. As data collection took place on school days, all recalls reflected dietary intake from the preceding weekday. The MyFood24 instrument utilizes standardized food portion photographs and common household measures to estimate the weight of consumed foods (26). For this study, the MyFood24 Middle East version was employed, which is specifically adapted to reflect dietary patterns in the region, including typical Kuwaiti foods, common cooking methods, and frequently consumed local food brands.

Trained researchers conducted face-to-face interviews with participants to obtain dietary information. Interviews followed a standardized protocol to ensure consistency in data collection and food item entry into the MyFood24 system. The resulting dietary output provided detailed estimates of energy intake, as well as macro- and micronutrient content for each reported food item consumed by the participants.

### Diet quality assessment

Diet quality was assessed using the Children’s Eatwell Guide (C-EWG) score, which evaluates adherence to dietary recommendations across eight food and nutrient components: total fat, saturated fat, free sugars, dietary fiber, salt, fruit and vegetables, fish, and red/processed meat (27). Each component was scored using a binary system: 1 point was assigned if the participant met the recommended intake, and 0 points if the recommendation was not met. The total C-EWG score ranged from 0 to 8, with higher scores indicating greater adherence to the dietary guidelines and, consequently, better overall diet quality.

### NOVA classification

We employed the NOVA classification system to categorize 487 study-specific food and beverage items into one of four groups (NOVA 1 to NOVA 4), based on the extent and purpose of industrial processing (10). As the NOVA system requires detailed ingredient information for accurate classification, the absence of such data, particularly for complex food items, necessitated certain assumptions. In cases where ingredient information was unavailable, items described as homemade were classified as processed foods (NOVA 3), while those identified as retail or commercially prepared were classified as UPF (NOVA 4).

Our primary variable of interest was the intake of UPFs, which was assessed using two metrics: the percentage of total energy intake derived from UPFs (%kcal) and the absolute weight of UPFs consumption (grams/day).

### Covariates

The primary outcome variables were the C-EWG score and the percentage of total energy intake from macronutrients (carbohydrates, protein, fat, and saturated fatty acids), along with the intake of trans-fatty acids (TFAs), dietary fiber, and salt, all expressed in grams per day.

We collected height and weight to measure participants’ body mass index (BMI). BMI was classified into four categories (Underweight < 5th percentile, Normal 5th–85th percentile, Overweight or obese 85th percentile, Obese ≥ 95th percentile), based on the 2022 CDC BMI-for-Age and sex-specific. Whereas waist circumference (WC) was categorized by the Kuwait Age- and Gender-Specific WC Percentiles (28), WC ≥ 90th percentile was classified as a metabolic risk WC (29).

### Statistical analysis

All statistical analyses were conducted using R software (version 3.6.1), with statistical significance set at *P* < 0.05. The continuous variables (total energy intake from UPFs and the absolute weight of UPF consumption) were assessed for normality using the Shapiro–Wilk test and found to be non-normally distributed. Consequently, non-parametric tests were employed to examine differences in intake across the study variables. Descriptive analyses were conducted to report the prevalence of each categorical variable within the study sample, including nationality, governorate, smoking status, WC, and BMI categories.

To assess the effect of UPFs on overall diet quality and nutrient intake, we built eight linear regression models, one for each dependent variable. Our dependent variables were the C-EWG score and the percentage of total energy intake from macronutrients (carbohydrates, protein, fat, and saturated fatty acids), along with the intake of TFAs, dietary fiber, and salt. Our main exposure variables were total energy intake from UPFs and the absolute weight of UPF consumption. All models were adjusted for sex, age, residence, and smoking status. Regression models were adjusted for total energy intake to assess the effect of UPF intake on dietary variables independently of energy intake. This approach is consistent with recent studies in adolescent populations [Chavez-Ugalde et al. (22)], though alternative approaches such as the residual method are also valid (22). Additionally, to address potential heteroskedasticity, robust standard errors (HC3) were applied.

## Results

Table 1 presents the characteristics of the study sample (*n* = 375), stratified by sex. The mean age of participants was 15.6 years. Females comprised a slightly larger proportion of the sample (53.1%, *n* = 199), and the majority of participants were of Kuwaiti nationality. The overall prevalence of overweight and obesity was 44.5%, with a slightly higher rate observed among females. Metabolic risk WC was slightly higher among females as well. In contrast, the prevalence of smoking was higher among males.

**TABLE 1:** Study sample characteristics.

Characteristics	All (375) *n* (%)	Female (*n* = 199) *n* (%)	Male (*n* = 176) *n* (%)
Nationality			
Kuwaitis	353 (94.1%)	187 (94.0%)	166 (94.3%)
Non-Kuwaitis	22 (5.9%)	12 (6.0%)	10 (5.7%)
Governorate			
Alahmadi	91 (24.3%)	56 (28.1%)	35 (19.9%)
Alasema	53 (14.1%)	35 (17.6%)	18 (10.2%)
Alfarwaniya	72 (19.2%)	29 (14.6%)	43 (24.4%)
Aljahra	74 (19.7%)	35 (17.6%)	39 (22.2%)
Hawali	46 (12.3%)	31 (15.6%)	15 (8.5%)
Mubarak AlKabeer	39 (10.4%)	13 (6.5%)	26 (14.8%)
Smokers			
No	317 (84.5%)	196 (98.5%)	121 (68.8%)
Yes	58 (15.4%)	3 (1.5%)	55 (31.2%)
BMI categories^a^			
Underweight	30 (8.0%)	15 (7.5%)	15 (8.5%)
Normal	178 (47.5%)	90 (45.2%)	88 (50.0%)
Overweight and obese	167 (44.5%)	94 (47.2%)	73 (41.5%)
Waist circumference category^b^			
Normal	245 (65.3%)	119 (31.9%)	125 (33.5%)
At metabolic risk	130 (34.7%)	78 (20.9%)	51 (13.7%)

^a^BMI categorized by the 2022 CDC BMI-for-Age and sex-specific.

^b^Waist circumference categorized by the Kuwait Age- and sex-specific waist circumference percentiles (28).

### Differences in% energy from UPFs and grams of consumption of UPFs

Table 2 presents differences in the percentage of energy intake and gram consumption of UPF across various sample characteristics. Significant sex differences were observed, with females consuming a higher percentage of energy from UPF (48.3%) but lower absolute UPF consumption in grams (377.0 g) compared to males (39.3% and 554.5 g, respectively; *p* = 0.007 and *p* < 0.001).

**TABLE 2:** Differences in% energy from UPF^a^, and grams of consumption of UPF across sample characteristics.

Characteristics	% energy from UPF Median (IQR)	*P*-value	Grams of consumption of UPF Median (IQR)	*P*-value
Total	43.9 (45.7)		443 (490.3)	
Sex		0.007*		6.31e-06*
Female	48.3 (41.8)		377.0 (522.0)	
Male	39.3 (47.8)		554.5 (514.7)	
Nationality		0.54		0.87
Kuwaitis	43.9 (43.9)		446.0 (497.0)	
Non-Kuwaitis	45.9 (67.8)		402.5 (379.8)	
Governorate		0.21		0.02*
Alahmadi	36.4 (35.4)		401.0 (456.5)	
Alasema	43.4 (37.7)		370.0 (606.4)	
Alfarwaniya	52.9 (49.9)		602.0 (604.0)	
Aljahra	41.9 (44.4)		505.0 (421.0)	
Hawali	48.7 (45.2)		324.5 (386.5)	
Mubarak AlKabeer	43.7 (49.5)		391.3 (417.9)	
Smokers		0.10		0.002*
No	45.4 (45.5)		414.0 (467.5)	
Yes	40.9 (46.9)		629.0 (567.5)	
BMI categories^b^		0.74		0.16
Normal	44.9 (45.3)		463.0 (490)	
Overweight and obese	43.0 (44.0)		408.0 (515)	
Waist circumference category*^c^*		0.24		0.03*
Normal	43.0 (48.4)		482.5 (513.7)	
At metabolic risk	46.2 (43.4)		362.0 (531.3)	

*P-value < 0.05, Kruskal-Wallis and Mann-Whitney test.

^a^Ultra-processed food (UPF), food items were classified by NOVA classification system (10).

^b^BMI categorized by the 2022 CDC BMI-for-Age and sex-specific.

*^c^*Waist circumference categorized by the Kuwait Age- and sex-specific waist circumference percentiles (28).

No significant differences in UPF intake were found by nationality. However, UPF consumption varied significantly across governorates in grams (*p* = 0.02), with participants from Alfarwaniya reporting the highest intake (602.0 g) and those from Hawali the lowest (324.5 g).

While smokers did not differ significantly in% energy from UPF (*p* = 0.10), they consumed significantly more UPF in grams than non-smokers (629.0 vs. 414.0 g; *p* = 0.003).

No significant differences in UPF intake were observed across BMI categories. However, participants classified as having a WC indicating metabolic risk consumed significantly less UPF in grams compared to those with a normal WC (362.0 vs. 482.5 g; *p* = 0.03), despite no significant difference in percentage energy intake.

### Distribution of ultra-processed food intake categories among Kuwaiti adolescents by sex

**FIGURE 1:**
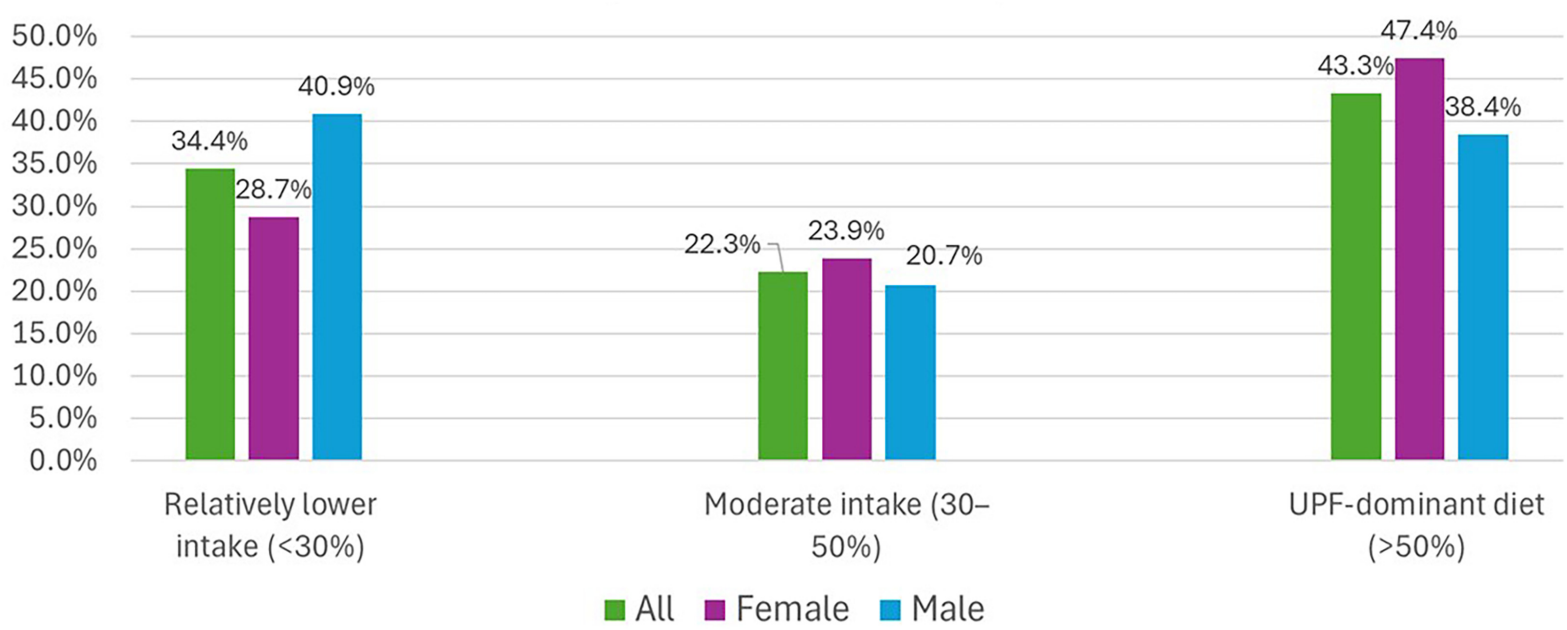
Distribution of ultra-processed food (UPFs) intake categories among Kuwaiti adolescents by sex.

The distribution of UPF intake categories among Kuwaiti adolescents differed slightly by sex. Overall, 34.4% of adolescents fell into the “Relatively lower intake” category (<30% of total energy from UPFs), 22.4% into the “Moderate intake” category (30%–50%), and 43.2% into the “UPF-dominant diet” category (>50%) (30). Among females, 28.7% were in the lower intake category, 23.9% in the moderate intake category, and 47.3% in the UPF-dominant category; among males, these proportions were 40.9%, 20.7%, and 38.4%, respectively. A Chi-square test suggested a borderline difference in the distribution between sexes (*p* = 0.056). The distribution of these categories is presented visually in Figure 1.

#### The effect of% energy from ultra-processed food on diet quality and nutrient intake

Table 3 presents the results of linear regression analysis examining the associations between the percentage of energy from UPF (% energy from UPF) and various measures of diet quality and nutrient intake. No significant association was found between% energy from UPF and the overall C-EWG (*B* = −0.001; 95% CI: [−0.004, 2.86]) or carbohydrate intake (*B* = 0.006; 95% CI: [−0.03, 0.04]).

**TABLE 3:** Linear regression analysis for diet quality and nutrients association with% energy from ultra-processed food^a, b^.

Dietary characteristics	B	*P*-value	95% CI
Children Eat Well Score	−0.001	0.59	[−0.004, 2.86]
% energy from carbohydrates	0.006	0.78	[−0.03, 0.04]
% energy from protein	−0.07	<0.0001*	[−0.10, −0.04]
% energy from fat	0.08	<0.0001*	[0.05, 0.12]
% energy from saturated fatty acids	0.03	0.001*	[0.07, 0.05]
Total trans fatty acids intake (grams)	0.005	0.01*	[0.0009, 0.01]
Total dietary fiber intake (grams)	−0.5	<0.0001*	[−0.62, −0.37]
Total salt intake (grams)	0.03	<0.0001*	[0.02, 0.05]

*P < 0.05.

^a^Ultra-processed food (UPF), food items were classified by the NOVA classification system (10).

^b^Model adjusted for age, sex, nationality, governorates, smoking status, and total energy intake.

However, higher% energy from UPF was strongly and significantly associated with lower protein intake (*B* = −0.07; 95% CI: [−0.10, −0.04]) and higher fat intake (*B* = 0.08; 95% CI: [0.05, 0.1]). Similarly, saturated fatty acid intake was positively associated with% energy from UPF (*B* = 0.03; 95% CI: [0.07, 0.05]). Total TFAs intake was also significantly positively associated with% energy from UPF (*B* = 0.005; 95% CI: [0.0009, 0.01]). Total dietary fiber intake showed a negative association with% energy from UPF (*B* = −0.5; 95% CI: [−0.62, −0.37]). Finally, salt intake was positively associated with% energy from UPF (*B* = 0.03, 95% CI: [0.02, 0.05]).

To make these results more interpretable, we examined the effect of increasing UPF intake from the 25th to the 75th percentile within the sample. This increase was associated with a 0.7% decrease in protein intake, 0.8% increase in total fat, 0.3% increase in saturated fatty acids, a 0.003 g increase in TFA intake, and a 0.4 g increase in salt intake. These findings indicate that higher UPF consumption is linked to less favorable nutrient profiles and higher intake of nutrients associated with adverse health outcomes.

#### The effect of ultra-processed food (grams/day) on diet quality and nutrient intake

Table 4 presents the results of a linear regression analysis examining the association between UPF (grams/day) and diet quality, along with nutrient intake. The analysis revealed that higher UPF consumption was significantly associated with poorer diet quality, as indicated by a lower C-EWG (*B* = −0.002; 95% CI: [−0.0004, −0.0008]).

**TABLE 4:** Linear regression analysis for diet quality and nutrients association with ultra-processed food consumption defined as (grams/day)^a, b^.

Dietary characteristics	B	*P*-value	95% CI
Children Eat Well Score	−0.002	0.04*	[−0.0004, −0.0008]
% energy from carbohydrates	0.001	0.95	[−0.002, 0.004]
% energy from protein	−0.001	0.09	[−0.002, 0.0001]
% energy from fat	0.002	0.06	[−0.0001, 0.004]
% energy from saturated fatty acids	−0.0009	0.12	[−0.003, 0.001]
Total trans fatty acids intake (grams)	0.0009	<0.0001*	[0.0005, 0.0012]
Total dietary fiber intake (grams)	−0.004	<0.0001*	[−0.007, −0.002]
Total salt intake (grams)	0.004	<0.0001*	[0.003, 0.005]

*P < 0.05.

^a^Ultra-processed food (UPF), food items were classified by the NOVA classification system (10).

^b^Model adjusted for age, sex, nationality, governorates, smoking status, and total energy intake.

Additionally, intake of total TFAs (*B* = 0.0009; 95% CI: [0.0005, 0.0012]) and total salt intake (*B* = 0.004; 95% CI: [0.003, 0.005]) were positively and significantly associated with UPF consumption. Conversely, total dietary fiber intake was inversely associated (*B* = −0.004; 95% CI: [−0.007, −0.002]), suggesting that higher fiber intake corresponds to lower consumption of UPF.

To make these results more interpretable, we examined the effect of increasing UPF consumption from the 25th to the 75th percentile within the sample. This increase was associated with a 2.8-point decrease in the C-EWG score, a 0.09 g increase in TFA intake, a 0.4 g decrease in dietary fiber intake, and a 0.4 g increase in salt intake. These findings indicate that higher absolute UPF consumption (grams/day) is associated with poorer overall diet quality, lower fiber intake, and higher intake of nutrients linked to adverse health outcomes.

## Discussion

In this cross-sectional study of Kuwaiti adolescents, we found that the median intake of UPFs was 443 g/day, contributing 43.9% of total daily energy intake. After adjusting for age, sex, and total energy intake, the percentage of energy derived from UPFs was inversely associated with protein and fiber intake, but positively associated with total fat, saturated fat, and TFAs. A higher consumption of UPFs (by weight) was linked to poorer overall diet quality, characterized by lower fiber intake and higher intakes of salt and TFAs. These findings highlight the substantial contribution of UPFs to adolescents’ diets in Kuwait and their potential adverse impact on nutritional quality.

To our knowledge, this is the first study to assess the intake of UPFs among adolescents in Kuwait, making it difficult to evaluate temporal changes in dietary behavior within this group. While the NOVA classification system is widely used to categorize foods by degree of processing, it has been noted that not all UPFs are inherently unhealthy, and some critics highlight conceptual boundaries of the system. In this study, NOVA provides a consistent framework for assessing the contribution of UPFs to adolescent diets in Kuwait (31). Evidence from other countries indicates that UPF consumption is increasing globally, with notable differences between upper-middle- and high-income countries (32, 33). A meta-analysis by Neri et al. across eight countries reported that the proportion of total energy intake from UPFs among adolescents ranged from 19% to 36% in upper-middle-income countries such as Argentina, Brazil, Colombia, and Mexico, and from 34% to 68% in high-income countries such as Australia, Chile, the United States, and the United Kingdom (34). Given that Kuwait is classified as a high-income country, our finding that UPFs contribute 43.9% of total energy intake among adolescents aligns with patterns observed in other high-income settings. Our findings are also consistent with emerging evidence from the Middle East and other regions showing high consumption of UPFs among young populations. For example, a study among children and adolescents in Jordan reported that UPFs contributed approximately 40% of total energy intake and were associated with obesity indicators and waist circumference (35). Similar concerns have been reported in the Gulf region, where higher UPF intake in Saudi populations has been linked with higher BMI and abdominal obesity (36).

Several factors could contribute to this prevalence in UPF intake, in high-income countries and in Kuwait. First, Kuwait’s high-income context has fostered a “nutrition transition”: characterized by energy-dense, nutrient-poor foods that are widely available at low cost, while fiber and micronutrient intake remain low (37, 38). Second, lifestyle changes and urbanization are favoring convenience, ready-to-eat meals, fast-food outlets, and heavily processed items, and decreasing the time for home cooking (35). Third, gaps in nutrition literacy and food-choice skills among adolescents may limit their capacity to navigate processed food marketing and make healthier choices (39). Fourth, the prevalence of Westernized dietary patterns, aggressive marketing of UPFs, and cultural adoption of fast food and snack consumption likely further drive the shift away from traditional diets. Overall, these interlocking influences create an environment in which UPF consumption becomes a default dietary pattern for many adolescents in Kuwait.

Our findings suggest that UPF consumption (weight) is a significant predictor of low C-EWS, which reflects that the overall diet quality among adolescents in Kuwait is negatively affected by their consumption of UPF. Studies consistently show that poor overall diet quality is associated with a higher risk of obesity among adolescents. For example, U.S. data found lower Mediterranean Diet adherence linked to higher odds of obesity (40), and New Zealand adolescents with poorer diet quality had greater body-fat percentage and fat-to-lean mass ratio (41). Also, studies suggested that high diet quality was linked with reduced risk of metabolic syndrome in children and adolescents (42). Given this evidence, the high consumption of UPFs among Kuwaiti adolescents may further decline overall diet quality, thereby heightening their vulnerability to obesity and related metabolic disorders. This concern is particularly pressing as Kuwait already reports one of the highest adolescent obesity prevalence rates in the region (43), suggesting that continued high UPF intake could escalate existing public health challenges.

Similar to other findings, we observed that UPF consumption (%TEI and weight) is positively associated with total energy from fat, SFA, and total TFAs intake. Several studies have reported similar associations among adolescents, showing that higher UPF intake correlates with increased consumption of total and saturated fats and TFAs (44, 45). This pattern reflects the typical nutrient profile of UPFs, which are often energy-dense and rich in unhealthy fats. Such nutrient imbalances can contribute to excess energy intake and metabolic dysregulation, ultimately increasing the risk of obesity and related non-communicable diseases. Especially, TFAs are associated with increased risk of obesity and adverse metabolic profiles among children and adolescents. For example, Spanish and Japanese studies linked higher TFA intake to greater overweight/obesity and higher WC or HbA1c (46, 47).

Dietary fiber and protein intake were negatively influenced by UPF consumption among adolescents in Kuwait. This was consistent with previous findings, where higher UPF intake was associated with lower consumption of fruits, vegetables, whole grains, and lean protein sources (11). Sustained consumption of UPFs among adolescents in Kuwait and other countries may displace the intake of nutrient-dense foods, including fruits, vegetables, and whole grains, thereby reducing overall dietary fiber intake. This reduction in fiber is associated with impaired satiety regulation, higher energy consumption, and an increased risk of obesity and related metabolic disorders during adolescence (44, 48).

### Public health recommendation

These findings highlight the urgent need for targeted public health interventions to improve adolescent diet quality in Kuwait. Currently, comprehensive school nutrition policies or regulatory measures targeting UPFs consumption among adolescents are limited. This represents an opportunity for national authorities, including the Ministry of Health, the Ministry of Education, and relevant food safety agencies, to develop actionable strategies. Possible measures could include establishing school-level nutrition committees to monitor canteen offerings, restricting the availability and marketing of UPFs to students, integrating structured nutrition education programs into the curriculum, and engaging parents and caregivers to support healthy dietary patterns at home. Lessons from other countries suggest that interventions such as front-of-pack labeling, taxation of sugar-sweetened beverages, and restrictions on marketing of unhealthy foods to children can be effective and could inform the development of context-appropriate strategies in Kuwait.

### Strengths and limitations

To our knowledge, this is the first study to assess the consumption of UPF and its relationship with nutrient intake and dietary Patterns in adolescents in Kuwait, filling an important gap in regional public health research. We employed 24-h dietary recalls to collect dietary intake data. This method has been previously shown to have low random error and bias and high precision, making it a reliable tool for dietary assessment in population-based studies (49).

Although this study has several strengths, it is not without limitations. First, the cross-sectional design limits the ability to establish causal relationships between UPF consumption and dietary intake. Second, important environmental and sociodemographic factors that may influence dietary behaviors, such as household income, parental education, physical activity levels, sedentary behaviors (e.g., screen time), and the school or household food environment, were not captured in this study (50). The absence of these variables in the regression models may result in residual confounding, which should be considered when interpreting the observed associations. These variables could provide additional context for understanding adolescent food choices in Kuwait. In addition, information on the source or location of food consumption (e.g., home, school canteens, or external food vendors) was not collected, which limits the ability to contextualize where UPFs are primarily obtained or consumed among adolescents in Kuwait. Although students were sampled within schools, clustering could not be modeled due to the absence of school identifiers; therefore, robust standard errors (HC3) were used, though some residual clustering may remain. Finally, the use of a single 24-h dietary recall provides only a snapshot of intake and may not fully reflect habitual dietary behaviors, particularly given the variability in adolescent eating patterns.

## Conclusion

The results of this study indicate that higher consumption of UPFs is associated with a nutrient profile characterized by lower protein and fiber intake and higher intakes of fat, saturated fat, TFAs, and salt. This pattern promotes nutrient imbalances linked to an increased risk of obesity and related metabolic disorders, which is particularly concerning given the already high prevalence of adolescent obesity in Kuwait. These findings underscore the urgent need for targeted public health strategies to encourage healthier eating behaviors and reduce the long-term burden of diet-related non-communicable diseases in this population.

## Data Availability

The raw data supporting the conclusions of this article will be made available by the authors, without undue reservation.
